# Examining the impact of university-industry collaborations on spin-off creation: Evidence from joint patents

**DOI:** 10.1016/j.heliyon.2023.e19533

**Published:** 2023-08-25

**Authors:** Hugo Martínez-Ardila, Ángela Castro-Rodriguez, Jaime Camacho-Pico

**Affiliations:** a Universidad Industrial de Santander-UIS, Escuela de Estudios Industriales y Empresariales - EEIE, Research Group on Management of Technological Innovation and Knowledge – Innotec, Cra 27 Calle 9, Bucaramanga, Santander, Colombia; b REDDI- Technological Development Agency; Cl 8 #3-14, San Pedro, Valle Del Cauca Cali, Colombia

**Keywords:** University spin-off creation, University-industry collaborations, Previous collaborations, Technology transfer

## Abstract

The literature on entrepreneurship and technology transfer highlights several factors that impact the creation of university Spin-Offs. However, there is a limited body of research that specifically explores the impact of university-industry collaborations on the performance creation of these spinoffs. This study aims to fill this gap by examining the effects of university-industry collaborations on the creation of Spin-Offs from two perspectives: the number of university collaborations with different companies and the number of previous collaborations between the same university-industry dyad. The research employs joint patents as a source to measure the university-industry collaborations and statistical methods to empirically examine the impact of these collaborations on Spin-Off creation. The study is based on data from 108 universities between the years 2014 and 2017. The findings of this study reveal that both the number of collaborations and specially the presence of previous collaborations between the university and industry have a positive effect on the creation of Spin-Offs. These results suggest that universities and companies should consider these findings when formulating their strategies or policies for technology transfer and innovation management by encouraging university-industry collaborations.

## 1 Introduction

The third mission of universities, known as extension or projection, which includes the transfer of knowledge, has gained great relevance in recent years. This is because it is considered a key element that impacts economic development in countries [1]. It is worth noting that spin-offs, also referred to as entrepreneurial spin-offs or centrifugal venture generation, are one of the main drivers of direct commercialization of university intellectual property. They are also a source of local and national economic growth and have the ability to provide significantly higher revenues to universities than patent licensing [2].

In light of the importance of spin-offs, research has been conducted to identify the factors that influence the successful creation of university spin-offs [[3], [4], [5], [6], [7], [8]]. This research has focused on organizational determinant issues such as the role of the Technology Transfer Office (TTO), the characteristics of the university and of the professors-researchers, the university location, and to a lesser extent on university-business relations as a key factor that could translate, not only in greater entrepreneurial capabilities of the academic community, but also in a greater number of spin-offs created.

This study aims to contribute to the debate on the factors that influence the creation of university Spin-Offs, with a focus on those that would lead to the generation of this transfer mechanism from university-industry collaborations. Specifically, unlike previous research that has focused on identifying the main characteristics of universities and academics that promote the creation of spin-offs [[9], [10], [11], [12], [13], [14], [15], [16], [17]], the present research studies the relational aspects of university-company collaborations, referring to the number of collaborations that universities have with different companies and the previous relationships between the same university-company dyad, and the effect that these two variables have on the creation of new technology-based companies.

More specifically, although research literature argue that the relationship between academics and industry generates an impact on Spin-Off creation [18], that closer alliances with industry result in higher levels of commercialization [19,20], that cooperation with industry agents helps to the internationalization of the Spin-Off [21], and that such communication channels and networks generated from University-Industry collaboration benefit the industrialization of research accomplishments [22], there is a gap in the literature of University-Industry collaborations as a determinant factor that is directly related and measured to the creation of universities Spin-Offs. This is of particularly interest when there is a high load of theoretical information about the factors (e.g. previous collaborations) that influence the ability of an academic researcher to commercialize research [23], making the empirical analysis a critical step in the research domain. This study provides an original contribution to the existing literature by delivering a more comprehensive view of the phenomenon by assessing not only the effect of university-industry collaboration on the creation of university spin offs, but also the temporal effect of repeated or prior collaborations between the same dyad University-industry-, analyzing joint patents as the reflection of the collaboration between universities and companies.

To address the study of these variables, this article is organized as follows. In section 2, the study conducts the literature review and present the hypotheses arguments about university-company collaborations to identify those variables that influence the creation of university spin-offs as a mechanism for technology transfer. Section 3 describes the data, study sample, databases, tools, and methods used in the research empirical analysis. Section 4 presents the results, and section 5 the discussion and conclusion. This last section also describes the limitations and future research direction.

## 2 Literature review and hypothesis

### 2.1 University spin-offs

University Spin-Offs, also known as academic Spin-Offs, have been widely studied in the literature of entrepreneurship and innovation management. The concept is typically defined as a new company that is founded by a university, a member of the academic community, or an investor, and is created to commercially exploit knowledge or technology owned by the university through the production of goods and services [[24], [25], [26]].

However, there are variations in the definitions of University Spin-Offs in the literature. Some studies include the exploitation of any piece of intellectual property of the academic institution, such as brands, industrial designs, industrial secrets, and copyrights [4]. Others focus on the central technology or technological innovation that the new firm is founded around [17]. Additionally, some definitions state that the licensing of the intellectual property of this new company is not only generated by universities, but also by public research institutes [14,27] or more generally, public research organizations [28].

The founders of University Spin-Offs can vary as well. They can be employees of the university [17] or academics such as professors, students, or members of the university [29,30]. However, it is also acknowledged that the members of the company are not necessarily academics but inventors or investors that could or not be currently affiliated to the academic institution [31].

Furthermore, the mechanism of technology or technological innovation transfer is often emphasized in the literature to explain the direction of knowledge flow from the academic institution to the new formed company [17,32].

Given the variations in the definitions of University Spin-Offs in the literature, it is important for this research to establish a clear and specific definition that aligns with the objectives and scope of the study. Thus, for the purposes of this study, a University Spin-Off is defined as a new company founded by a university, a member of the academic community, or an investor, and created to commercially exploit knowledge or technology owned by the university through the production of goods and services.

### 2.2 University-industry collaborations

University-industry collaborations, also known as university-industry partnerships, refer to the interaction between academic institutions and industry organizations to foster knowledge and technology exchange [33,34], even if these interactions are made via an intermediary agent [35]. These collaborations have gained significant attention in recent years as a means of promoting technological advances and economic competitiveness of regions. Researchers have focused on understanding the nature and importance of these collaborations, particularly the mechanisms that facilitate knowledge transfer [20].

Three elements are considered to define the relational context of the transfer [36]. The first is trust, a key factor in the relational vision of alliances [37]. Trust can be measured through the existence of previous alliances or collaborations between the partners. The second, is the intensity of ties, understood as the frequency of contacts and communications, which promotes the degree of familiarity and reciprocity between the parties. Intensity of ties can be measured similarly like trust, by previous participation in cooperative alliances. The third corresponds to the distances that exist between the parties or partners. Distance involves not only geographic or physical distance, but other kind such as organizational distance, knowledge base distance or technological distance, cultural distance, and normative distance. Therefore, the relational capital is a critical driver of organizational knowledge transfer in this network of relationships [38].

Mansfield [39] demonstrated that over 10% of new products and processes introduced by large firms in various industries were dependent on academic research. Skute et al. [40] proposed three arguments for the study of university-industry collaborations: 1) industries are increasingly engaging in collaborative activities with academic institutions to gain knowledge and access to research and product development capabilities, 2) the quality of partners in the collaboration is crucial for successful innovation processes, and 3) policymakers recognize university-industry collaborations as a tool to address social and economic challenges of regions and enhance their innovation capacity [22].

University-industry collaborations can take various forms, including joint ventures, networks, consortia, and alliances [41]. These forms vary according to the degree of engagement and involvement of the participants. For example, joint ventures refer to a formal partnership between a university and a company to jointly develop a specific project or technology, while networks refer to a less formal arrangement where the parties share information and resources but retain a certain degree of autonomy.

### 2.3 University-industry collaborations and the creation of university spin-offs

University-Industry collaborations have been widely recognized as an important source of new knowledge and R&D, and have become a key mechanism for fostering innovation and economic competitiveness. Furthermore, University-Industry collaborations have been identified as an important factor in the creation of University Spin-Offs, as they provide opportunities for academics to develop and co-develop inventions that can be protected and commercialized as an entrepreneurial opportunity [33,42].

Besides key internal activities such as entrepreneurship education [43] and team composition [44], previous studies have shown the effect of external agents as companies on the creation of university spin offs to commercialize research findings [45]. For instance, industry tends to fund less risky research than government funds, and then University-Industry interactions may affect technology transfer processes more directly than University-Government interactions [4,46]. Furthermore, it has been found that university scientists who collaborate with industry, receive funding from industry, or possess industry experience are more inclined to start companies [[47], [48], [49]].

Moreover, it has been suggested that the provision of industrial funding in academic research positively affects the development of patents and Spin-Off companies [4,19]. Thus, it has been found that faculty members with long academic careers and few interactions outside the university tend to lack the business perspective and experience necessary to create a company [47,50]. These findings support the idea that University-Industry collaborations and the provision of industrial funding are key factors in the creation of University Spin-Offs.

In general, the literature suggests that University-Industry collaborations play an important role in the creation of University Spin-Offs, providing opportunities for the commercialization of knowledge and technology, and fostering the development of new businesses. However, more research is needed to understand the specific mechanisms through which these collaborations affect the creation of University Spin-Offs, as well as the impact of different forms of collaborations and funding on the success of these ventures.

### 2.4 Hypotheses

In the domain of university spin-offs, the key role of university-industry collaborations in the emergence of academic ventures, including spin-offs, is widely recognized [27,51]. While academic research is a necessary condition for creating business opportunities, it alone is insufficient to initiate the new venture process. Consequently, universities must acquire and cultivate market-related competencies to effectively frame the business idea [24]. Notably, university-industry collaborations have been shown to facilitate the generation of university spin-offs by enhancing the understanding of market potential and the development of appropriate business models [52]. This could explain why academics engaged in collaboration agreements with industry often become involved in commercially-oriented activities and pursue entrepreneurial endeavors [53]. Furthermore, research cooperation with private companies has been found to have a significantly positive effect on the propensity to start a business [48].

As a consequence, the influence of university-industry collaboration on spin-off creation can be attributed to the broader access to knowledge and resources, as well as the expansion of non-academic or business networks [50]. This network expansion helps academic entrepreneurs internalize market-oriented motivations, values, and practices that may not be readily available within an academic environment [5]. This notion is supported by evidence demonstrating that successful universities continuously cultivate networks that contribute to the generation of a higher number of spin-offs [54], and also that expanding researchers' networks is positively associated with spin-off creation [55].

Remarkably, the interaction between universities and industry enables the transfer of business experience and the accumulation of critical capabilities and competencies necessary for new venture creation [53] For instance, universities acquire competencies related to opportunity refinement through experience and industry interaction, as well as leveraging competencies enabling them to access resources from industrial partners and effectively communicate with investors [24].

Therefore, by actively engaging with diverse companies, universities can enhance their entrepreneurial ecosystem, access valuable resources, and develop the necessary capabilities to foster spin-off creation. Building upon these arguments, it is hypothesized that.

Hypothesis 1 *A greater interaction of the university with different companies increases the probability of university Spin-Off creation.*

In general, the mechanism of previous interactions or collaborations implies that the frequency of collaborations between organizations strengthens the links and fosters higher levels of trust, closeness, and understanding [56]. Link strength, an important factor studied in collaborative networking and knowledge transfer, encompasses various elements such as time, emotional intensity, trust, and reciprocal services that characterize the relationship [[57], [58], [59], [60]]. Increased trust, familiarity, and mutual understanding between organizations contribute to effective knowledge transfer and a greater willingness to commit to helping recipients understand new external knowledge [36,58,59,61]. Building trust often starts from existing relationships or repeated collaborations, as these foster a small community where each party is familiar with the other [62].

Within the context of repeated university-industry collaborations, which are indicative of high trust and partner reputation [63], uncertainty, information asymmetry, transaction costs, and appropriability risks inherent in university-generated knowledge can be mitigated and can facilitate the development of joint innovations [20,64]. Previous knowledge and experience gained from past collaborations with companies are crucial for identifying entrepreneurial opportunities based on scientific research in universities, such as spin-offs [65]. Consequently, certain types of university-industry collaborations, such as technology licensing agreements and joint ventures, have been positively associated with spin-off creation [54,66]. Additionally, university-industry collaborations can take other forms, such as networks, consortia, and alliances, and their strength is influenced by previous collaborations, thereby impacting the selection of future partners [33,67,68].

In summary, the literature suggests that previous University-Industry collaborations are positively associated with the creation of university Spin-Offs. Therefore, it is hypothesized that.

Hypothesis 2 Previous collaborations of the same university-firm dyad have a positive effect on the creation of university Spin-Offs.

## 3 Methods

This research aims to examine the impact of university-industry collaborations on the creation of academic spin-offs. Data on the number of academic spin-offs was obtained from the Statistics Access for Technology Transfer (STATT) database of the Association of University Technology Managers (AUTM) for the years 2014–2017. The STATT database provides historical information on various technology transfer activities such as licensing income, spin-offs, patent activity, research funding, and other technology transfer activities of universities. Joint patents are employed as a measure of collaboration between universities and industry. Patent data has been utilized in previous studies to evaluate the technology transfer and outcomes of university-industry collaborations [68], as it is a readily available and accessible data source [22], and a critical indicator of innovation performance and industrial technology development [20,69]. The four-year period is chosen with the objective of obtaining a more precise representation of the actual spin-off creation activity in universities, as opposed to one-year measures [49,70]. Although previous research has suggested an average delay of seven years between an academic invention and a real commercial application [39], this research considers a shorter period appropriate as the dependent variable in this study pertains to the creation of a company rather than product or technology milestones [49].

### 3.1 Data and sample

The present study utilizes data from the Association of University Technology Managers' (AUTM) Statistics Access for Technology Transfer (STATT) database. The initial dataset includes 184 universities from the United States, which serves as an example of a mature and successful case of university spin-off creation. The United States' legislation promotes an environment conducive to university entrepreneurship, and its evolution has allowed universities to develop strategies for linking with their surrounding environment becoming interactive centers for companies and other organizations seeking solutions and pioneering collaborative innovation [71].

To refine the sample, two routes were established. The first route involved selecting 33 universities based on their ranking in Reuters' report of the 100 most innovative universities in the world and excluding university systems with multiple institutions or campuses in a single annual observation. These top-ranked universities serve as benchmarks in innovation activities and have a sufficient set of relationships with the industrial environment [20]. The second route expanded the sample by selecting universities that reported data in the STATT database each year from 2014 to 2017, resulting in an additional 75 universities after filtering out university systems and universities that did not report spin-off creation data.

In both routes, information not reported in the STATT database was obtained by reviewing the universities' annual technology transfer reports and annual statistics on R&D and innovation activities on their websites. If the information was still not available, it was requested via e-mail. The final study sample includes 108 universities, both public and private, located in the United States that created 2386 university spin-offs during the years 2014–2017.

Joint patents were used as a measure of university-industry collaborations. Patents from the universities were collected in a time window of five years prior to the creation of a spin-off (2009–2016) using the Orbit Intelligence tool database. This time window was chosen based on previous research suggesting that the lifespan of partnerships is typically no longer than five years [20,68,72] and is considered sufficient time to assess the impact in technological sectors [73].

Patents are an open and available source of data from which joint collaborations between actors in R&D and innovation activities can be identified. Various studies make use of patents to assess technology transfer and measure innovation [20,22,68] In addition, if universities formally engage in joint R&D activities with firms, the outcome of this collaboration is measurable from patent indicators, where patents that are jointly assigned to both partners is a result of the contribution in the alliance [68].

In the process of patent search and collection for each university, a careful review was conducted to identify and adjust misleading information such as typing errors (e.g., assignee names), changes in universities' legal figures and names, and ownership of patents (e.g., some patents are registered to Technology Transfer Offices or foundations belonging to the universities). These elements were key in structuring the search strategy in the Orbit Intelligence patent database by assignee, in addition to defining input elements for the search by patent family, patent number and title in the time window. A patent family refers to a group of patent documents that are associated with the same invention or multiple inventions sharing a common aspect. These documents can be published at different times within the same country or across different countries or regions [74]. The utilization of patent families is crucial in patent analysis to eliminate any bias that may arise from counting or considering the same invention multiple times during data processing.

### 3.2 Data processing and analysis

In order to determine the joint patents between each university in the sample and companies, the Orbit Intelligence patent database was reviewed on a patent-by-patent basis. During this process, some patents were filtered for reasons such as: only the university being present, only the university and the inventor being present in the field of applicants, the development being made with another university or with foundations, laboratories, or research institutes of a university, and not including a company. Additionally, patents that were developed jointly with the government (e.g., US Navy, US National Institutes Of Health (NIH), US Department Of Health & Human Services) were also filtered, since the aim is to analyze university-industry collaborations without considering the government.

However, one of the challenges encountered during the search process was the presence of repeated patents. Therefore, the publication number, legal actions, and names of the patents were verified, and repeated patents were eliminated. For the companies registered as holders, the identification of affiliates or subsidiary firms was performed to unify the name of the organization and count them as a single company. In this sense, when both the organization and its affiliates or subsidiary companies are registered as patent holders, the organization is counted as a single entity.

After the application of the filtering criteria and data cleaning, the extraction and organization of the joint patents were carried out in a database for each of the 108 universities in the established time periods.

### 3.3 Variables and model specification

#### 3.3.1 Dependent variable

The dependent variable in this study is the number of Spin-Offs *(SpinOffs)* created at a university (*i*) in a given year (*t*), during the period from 2014 to 2017. The data for this variable were obtained from the AUTM-STATT database, in line with previous research [4,[12], [13], [14],^19^,^30^,^49^,^70^].

#### 3.3.2 Independent variables

The first independent variable in this study is University-Industry Collaborations (*UICollab*). This variable is measured as the count of the number of different companies that share at least one joint patent with a university (*i*) in the sample during the five years prior to the observation year (*t*) when the Spin-Off was created (Kim & Song, 2007). For example, for the year of observation 2014 (*t*), the count of collaborations of each university (*i*) with (*n*) number of different companies is collected during the time frame from 2009 to 2013 (*t-5*, *t-1*). This time window is chosen based on the assumption that knowledge capital depreciates considerably, losing most of its value in five years [20].

**Fig. 1:**
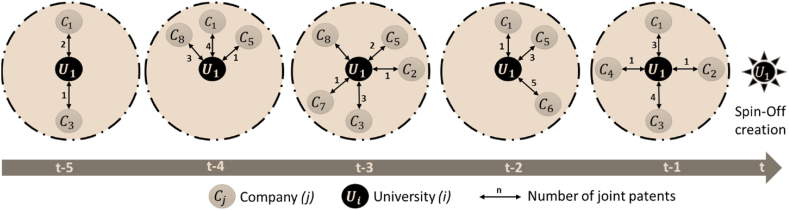
Example of University-Industry collaborations in a 5 year time window.

Fig. 1 illustrates an example of university-industry collaborations during the time frame of five years (*t-5*, *t-1*) prior to the year of observation or Spin-Off creation (*t*). In the figure, $U_{1}$ represents a university, $C_{j}$ represents different companies that have collaborations with the university, and the links or arrows depict these dyadic collaborations between the university and industry with the number of joint patents for each collaboration per year. As seen in Fig. 1, the university $U_{1}$ has eight (8) different companies, $C_{1}$ to $C_{8}$, with which it has different collaborations during the time window. Therefore, the variable university-industry collaboration (*UICollab*) is 8 for university $U_{1}$.

The second independent variable is Previous Collaborations (*PrevCollab*). This variable is measured as the number of dyads (*n*) between a university (*i*) and company (*j*), (*i*,*j*), that share at least two joint patents during the five years prior to the observation year (*t*) when the Spin-Off was created [75,76]. This variable was obtained by first collecting information on all university-industry collaborations (e.g. joint patents) for university (*i*) in the five-year time window. Next, an identification was made of the specific dyads of university (*i*) and company (*j*), (*i*,*j*), that had at least two joint patents during the five-year time window. To facilitate a comprehensive analysis of variations in the number of previous collaborations between universities and industries, the independent variable was categorized into three distinct levels using the Jenks classification method [77]. The utilization of this method guarantees an optimal arrangement of values into separate classes, minimizing the average deviation within each class while maximizing the deviation from the means of other classes. This approach enhances our understanding of the differences and enables a more nuanced examination of the variable's impact on the research outcomes.

#### 3.3.3 Control variables

To control for alternative factors that can explain the creation of university Spin-Offs, several control variables were included in this study.

##### 3.3.3.1 TTO experience (*TTOExper*)

Given that the capabilities of universities' technology transfer offices (TTO) may take some time to develop, the time that the TTO has been involved in technology transfer activities is considered a strong predictor of Spin-Off creation. This is because it represents previous experience and prior knowledge in technology transfer activities [12,19,29]. Therefore, to consider this knowledge and accumulated experience of a TTO, this variable *TTOExper* is defined as the natural logarithm of the years of operation of the TTO, measured as the difference between the year of observation and the year in which the university's TTO was founded.

##### 3.3.3.2 Patent application per capita (*PatentApp*)

Inventions capture the overall inventive activity at a university, regardless of whether those inventions are of interest to business [4]. In addition, one of the necessary conditions for the generation of Spin-Offs from universities is the availability of human capital such as scientists and engineers with the appropriate qualifications and know-how in R&D activities [49,78]. Therefore, this research controls for the university's patent application per capita that measures the relationship between the university's inventive capital and its human resources. Human capital in R&D activities in universities is defined as the number of main investigators and all other personnel who received salaries, wages, and fringe benefits for R&D activities according to data extracted from the Higher Education Research and Development Survey (HERD) database of the National Science Foundation (NSF). Accordingly, the variable *PatentApp* is measured as the natural logarithm of the count of total patent applications per university, divided by the university's human capital in each defined year.

##### 3.3.3.3 University reputation *(UnivReput)*

University reputation is defined as the intellectual eminence or prestige of the university [4,30,42] and can represent successful relationships of University-Industry collaborations [79]. Therefore, this variable *UnivReput* was measured according to the Academic Ranking of World Universities, compiled by the Institute of Higher Education at Shanghai Jiao Tong University. *UnivReput* is coded as a dummy variable, which assumes the value of one if the university is ranked in the top fifteen positions in the ranking according to the time range set for the research (2014–2017), zero otherwise [20].

##### 3.3.3.4 Technical orientation to medical school *(MedSchl)*

The technical orientation of a university is generally assessed based on the existence of a medical school [14,19,80]. In universities with a medical school, medicine is usually the field most served by the Technology Transfer Office [32] and medical inventions have greater marketability than other fields. Therefore, this variable is measured as a dummy variable, which assumes the value of one if the university has a medical school, zero otherwise.

In summary, this study includes several control variables to account for alternative factors that can explain the creation of university Spin-Offs. These include TTO Experience, Patent Application per Capita, University Reputation, and Technical Orientation to Medical School. These variables are measured using different techniques such as natural logarithm of years of operation, counts per capita, and dummy variables. The inclusion of these control variables allows for a more comprehensive analysis of the relationship between university-industry collaborations and the creation of Spin-Offs, accounting for any potential confounding factors.

#### 3.3.4 Model estimation and specification

This study uses a regression model to estimate the relationship between university-industry collaborations, measured by joint patents developed between the university and the firm, and the dependent variable (*SpinOffs*). Taking into consideration that the study design should align with the research question and hypotheses, this study employs a cross-sectional design to examine the relationship between university-industry collaborations and the creation of academic spin-offs at a specific moment in time. Additionally, the study aims to evaluate the characteristics of the population of universities in their spin-off creation process. Initially, the analysis focuses on the base year of 2014. However, to ensure robustness in the results and provide further evidence for the hypotheses, additional analyses were conducted for the years 2015, 2016, and 2017, adopting a repeated cross-sectional approach [81]. It is important to note that cross-sectional studies have limitations, as they cannot establish causality between variables or track changes in individual-level outcomes over time. Nevertheless, cross-sectional designs serve as a valuable starting point for research, offering insights and laying the groundwork for subsequent studies that can incorporate more sophisticated methodologies [82].

Two hypotheses were formulated in this study.

Hypothesis 1 posits that “greater university interaction with different firms increases the probability of university Spin-Off creation”. To test this hypothesis, a regression model was used as described in equation (1):

(1) $$SpinOffs=f_{n}\left(UICollab;\;Control\;variables\right)$$

where *SpinOffs* is the number of Spin-Offs created by university (*i*), which is a function of the number of different University-Company collaborations *UICollab* and the defined control variables.

Similarly, Hypothesis 2 states that “prior university-firm collaborations have a positive effect on the creation of university Spin-Offs”. To test this hypothesis, another regression model was used as described in equation (2):

(2) $$SpinOffs=f_{n}\left(PrevCollab;Control\;Variables\right)$$

where *SpinOffs* is the number of Spin-Offs created by university (*i*), which is a function of the number of previous University-Company collaborations *PrevCollab* and the defined control variables.

The basic regression model for count data is the Poisson regression model. However, a drawback of the Poisson distribution is the equidispersion, as typically the data show that the conditional variance almost always exceeds the conditional mean, or overdispersion [83]. Since the dependent variable is based on count data that takes non-negative and integer values, a first approach was the Poisson regression model. However, examining the distribution of the *SpinOffs* dependent variable as Poisson, a goodness-of-fit test rejected the Poisson distribution assumption due to overdispersion. Therefore, a negative binomial estimator was used as it is more appropriate for this data.

## 4 Results

This section presents the results of the study. The summary statistics of the variables including the correlation matrix are presented in Table 1. As expected, the data showed overdispersion, with the variance of the independent variable *SpinOffs* (31.584) being greater than its mean (5.52), supporting the use of negative binomial regression for overdispersed count data. The dependent variable *SpinOffs* has a high correlation with the independent variable *UICollab* (0.804), indicating a possible causality problem. However, the Granger causality test was conducted, and it rejected causality between the two variables with lags of three years.

**Table 1:** Descriptive statistics and correlation matrix.

	Mean	SD	1	2	3	4	5	6	7
1. SpinOffs	5.52	5.62	1						
2. UICollab	19.42	18.47	0.804***	1					
3. PrevCollab	6.07	7	0.653***	0.860***	1				
4. TTOExper	27.86	27.86	0.395***	0.417***	0.282***	1			
5. MedSchl	0.57	0.50	0.295***	0.287***	0.151***	0.138**	1		
6. PatentApp	0.03	0.03	0.331***	0.361***	0.385***	0.039	−0.054	1	
7. UnivReput	0.10	0.30	0.605***	0.628***	0.491***	0.226***	0.104**	0.401***	1

*p < 0.1; **p < 0.05; ***p < 0.01.

As expected, the independent variables are highly correlated, indicating a potential multicollinearity problem. To assess the existence of multicollinearity in the model, an evaluation of the variance inflation factor (VIF) and the condition index (CI) was conducted. The maximum value of the VIF was 2.364 with a mean value of 1.562, which is below the threshold of 10 [84]. Additionally, the maximum CI value in the model was 23.326, which is less than 30. Therefore, although some variables present high correlation, which is a necessary but not sufficient condition for the existence of multicollinearity, the tolerance of the VIF and CI values indicates that multicollinearity is not a threat to the model.

**Fig. 2:**
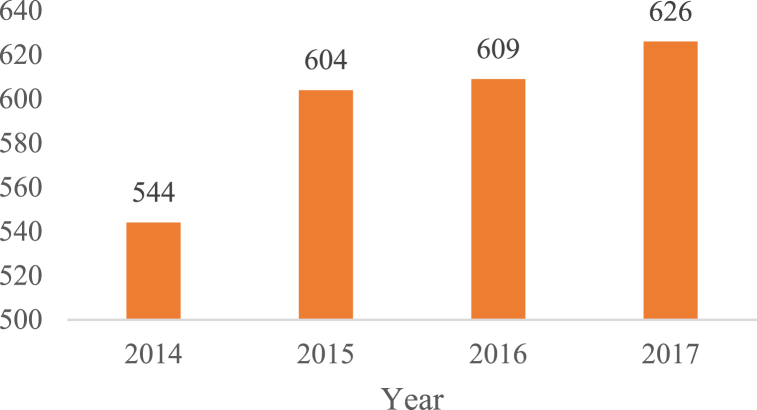
Number of University Spinoffs per year (2014–2017).

Fig. 2 illustrates the dynamics of university Spin-Off creation among 108 universities in the period from 2014 to 2017. According to the AUTM survey, 184 universities and university systems participated in this period, resulting in the identification of 3511 Spin-Offs formed. However, this study focuses on data derived from a subset of those academic institutions (108 universities) for which the dataset was available for the period of 2014–2017, with a total of 1068 Spin-Offs created.

In the US, spin-offs are a central focus of university technology transfer offices, as reported by the Association of University Technology Managers [85]. In 2021, 996 new university IP-based start-ups were formed which directly impacts local economies; two thirds of the new businesses are headquartered in their institution's home state. According to the AUTM survey, the majority of university startups are formed around patented technology, and this prolonged growth and higher survival rate reflect that startups with patents are 35 times more likely to succeed than startups without patents.

To estimate the impact of University-Industry collaborations on Spin-Off creation activity, this study utilized negative binomial regression models for each of the years 2014, 2015, 2016, and 2017. These models are reported in Table 2 and measure the number of new Spin-Offs created per year per university. A cross-sectional analysis was performed for each year to account for any changes in the size and statistical significance of the independent variables over the observation time period. For simplicity, Model 0 includes the university-level control variables as a baseline for year 2014. Models 1, 3, 5, and 7 evaluate the effect of the number of university joint collaborations with companies (*UICollab*). Models 2, 4, 6, and 8 evaluate the effect of previous Collaborations the university had with companies (*PrevCollab*), which has a 3-level factor, [0.5], (5.14], and (14.33], defined according to the Jenks' natural breaks classification method [77].

**Table 2:** Regression models per year (2014–2017).

			Dependent Variable: SpinOffs							
*Model*		(0)	(1)	(2)	(3)	(4)	(5)	(6)	(7)	(8)
Variables	*Year*	Base Model 2014	2014	2014	2015	2015	2016	2016	2017	2017
UICollab			0.035***		0.028***		0.029***		0.025***	
			(0.004)		(0.005)		(0.004)		(0.005)	
PrevCollab(5,14]				0.914***		0.781***		0.545***		0.528***
				(0.148)		(0.155)		(0.155)		(0.158)
PrevCollab(14,33]				1.273***		0.964***		0.748***		0.752***
				(0.227)		(0.221)		(0.202)		(0.206)
UnivReput		0.6116*	−0.335	0.120	−0.094	0.159	−0.162	0.312	0.085	0.448**
		(0.2419)	(0.206)	(0.206)	(0.230)	(0.224)	(0.205)	(0.219)	(0.236)	(0.212)
MedSchl		0.5879***	0.332**	0.501***	0.414***	0.511***	0.507***	0.584***	0.301**	0.400***
		(0.1644)	(0.131)	(0.138)	(0.142)	(0.143)	(0.127)	(0.140)	(0.136)	(0.140)
TTOExper		0.5595***	0.259*	0.585***	0.376**	0.562***	0.325**	0.521***	0.358**	0.543***
		(0.1685)	(0.132)	(0.145)	(0.148)	(0.149)	(0.136)	(0.150)	(0.155)	(0.156)
PatentApp		0.4443***	0.231**	0.208**	0.346***	0.392***	0.192**	0.326***	0.275***	0.340***
		(0.1104)	(0.091)	(0.100)	(0.102)	(0.104)	(0.094)	(0.101)	(0.103)	(0.106)
Constant		0.8950	0.529	−0.484	0.720	0.446	0.271	0.399	0.681	0.491
		(0.6600)	(0.507)	(0.604)	(0.563)	(0.598)	(0.541)	(0.607)	(0.609)	(0.639)
Observations		108	108	108	108	108	108	108	108	108
Log Likelihood		−262.181	−238.976	−242.789	−253.385	−255.720	−251.112	−261.381	−262.413	−267.580
theta		2.6741 (0.640)	7.818** (3.064)	5.791*** (1.894)	4.863*** (1.474)	4.591*** (1.404)	7.858** (3.077)	4.679*** (1.443)	4.925*** (1.446)	4.204*** (1.217)
AIC		536	490	500	519	525	514	537	537	549

Note: Columns 1, 3, 5 and 7 report the results of the estimates of the variable University-Company Collaborations (UICollab), and columns 2, 4, 6 and 8 report the estimates of the variable Previous Collaborations (PrevCollab) for the creation of university spin-offs in the years 2014, 2015, 2016, and 2017 respectively. *p < 0.1; **p < 0.05; ***p < 0.01. Standard errors in parentheses.

In Table 2, the Akaike information criterion (AIC) and the likelihood ratio test indicate an improvement in models including independent variables over the baseline model (Model 0) of year 2014. Similar behavior of AIC and likelihood ratio test improvement is found in each of the years 2015, 2016, and 2017. Additionally, the Wald test was used to evaluate the significance of the explanatory variables of the models and results indicate that both explanatory and control variables add significant explanatory power for all models.

The results of Model 1 indicate that the number of collaborations of a university with different companies (*UICollab*) has a significant positive influence on the number of university Spin-Offs created (β = 0.035, p < 0.01). This pattern is consistent in Models 3, 5, and 7 (β = 0.028, p < 0.01; β = 0.029, p < 0.01; β = 0.025, p < 0.01), respectively. These findings support Hypothesis 1, demonstrating the positive effect of a higher number of university collaborations with different companies on the probability of creating a Spin-Off. For example, a one-unit increase in the number of interactions with different companies is expected to result in a 0.035 unit increase in the expected counts of Spin-Offs created in the year 2014, while holding other variables constant.

Model 2 indicates that the number of previous collaborations with the same company (*PrevCollab*) has a significant positive effect on the number of Spin-Offs created, supporting Hypothesis 2. Specifically, the effect of having between 14 and 33 previous collaborations with the same company is significant and greater (β = 1.273, p < 0.01) than having between 5 and 14 previous collaborations (β = 0.914, p < 0.01). This pattern is consistent in Models 4, 6, and 8 (β = 0.964, p < 0.01; β = 0.781, p < 0.01; β = 0.964, p < 0.01; β = 0.781, p < 0.01; β = 0.964, p < 0.01; β = 0.781, p < 0.01), respectively. The estimated coefficients for *PrevCollab* imply that, ceteris paribus, having more than 14 previous collaborations has a greater effect on the number of Spin-Offs created than having between 5 and 14 previous collaborations.

It is important to note that the regression coefficients of the study variables in the models evaluated over the four years do not present significant variation, which not only supports the hypotheses put forward but also indicates that there is no unobserved individual heterogeneity that could be correlated with the regressors or bias in omitted variables.

Regarding the four control variables, the results of this study indicate that the presence of a medical school (*MedSchl*) in the university has a positive effect on the probability of creating a Spin-Off in all models, contrary to previous literature [14,19,80]. This finding is supported by previous research indicating that Spin-Offs tend to originate mainly in the sciences, especially in the field of life sciences such as biotechnology, pharmacology, and medical devices [[86], [87], [88]]. Furthermore, the study also found that the time of operation of the technology transfer office (*TTOExper*) has a positive effect on the probability of creating a Spin-Off in all models, likely due to the “learning effect” that occurs over time [70,89,90]. Additionally, the number of patent applications filed per year relative to the number of researchers in the university (*PatentApp*) also has a significant positive effect on the number of Spin-Offs created in all models.

On the other hand, the university reputation control variable (*UnivReput*) was only found to be significant in the baseline Model 0 of year 2014 (β = 0.6116, p < 0.05), and Model 8 at the year 2017 (β = 0.448, p < 0.05). However, it should be noted that when the variable *UICollab* is included in the base models each year, the statistical significance of the variable *UnivReput* changes, as well as the sign. For example, in the year 2014 in the base model, the variable *UnivReput* has a significant positive effect on the creation of Spin-Off (β = 0.6116, p < 0.05), but when including the variable *UICollab*, the regression coefficient of the variable *UnivReput* becomes negative and not significant (β = −0.335). Besides a possible collinearity between variables, these results suggest that when defining the creation of Spin-Off from the collaborations between the university and the external agents, the reputation of the university may not be as important as previously thought.

Overall, the results of this study provide insight into the factors that influence the creation of Spin-Offs in universities. The findings suggest that the presence of a medical school, the time of operation of the technology transfer office, and the number of patent applications are positively associated with the probability of creating a Spin-Off, while the university reputation may not be as important when taking into account collaborations with external agents. These results contribute to the current literature on university Spin-Offs and can inform policy decisions related to technology transfer and commercialization in universities.

## 5 Discussion and conclusions

This study aimed to investigate the importance of university-industry collaborations in the creation of university Spin-Offs. Using data from 108 selected universities, the results of this study suggest that university-industry collaborations play a significant role in the creation of new technology-based companies in universities. Specifically, the coefficients for our university-industry collaborations measurement models, which include the number of different collaborations accumulated in the five years prior to the Spin-Off creation and previous collaborations between the same university-industry dyad accumulated in the five years prior to the Spin-Off creation, showed positive and significant effects (p-levels <0.01).

Contrary to some research that argue that collaboration of some type of universities with industry seems to be not very significant for new companies' creation [8], the results support the hypotheses that a higher interaction of the university with different companies increases the probability of Spin-Off creation. The results are consistent with previous research in the field which claim that a high number of relationships with other companies and units increases the probability of access to new relevant knowledge, ideas, or potentially useful resources and therefore, increases the probability and the amount of organizational knowledge transfer [38]. In addition, Reagans and Mcevily [59] also suggest that relationships improve data processing capacity, which allows for a more effective knowledge flow; consequently, success in Spin-Off creation depends on both the type and size of the university's social network.

These findings highlight the significant importance of collaborations for companies when seeking opportunities for growth and innovation. Annique Un & Asakwa [91] classify R&D collaborations into four types based on their impact on process innovation, with collaborations involving universities demonstrating the second-highest impact, after the collaborations with suppliers. As such, organizations tend to prioritize collaborations with universities, which may be more conducive to spin-off creation. Collaborations with external partners, such as universities and research institutions, offer a particularly valuable avenue for companies to leverage academic expertise and research capabilities. These partnerships enable companies to bridge the gap between scientific research and practical applications, leading to the development of new products, services, and technologies [92,93]. The knowledge exchange and collaborative efforts between companies and universities can result in breakthrough innovations and novel solutions that would not have been possible through internal efforts alone [94].

Trust formation between university and industry partners is also crucial for the success or as a result of university-industry collaborations. Bstieler et al. [95] found a positive relationship between the flexibility and transparency of university intellectual property (IP) policies, shared governance by university-industry partners, and trust formation. Furthermore, the activities of university-industry champions amplify the positive effects of shared governance, reducing the significance of university IP policies in trust formation. Then, trust between partners is positively associated with knowledge transfer and innovation performance. This underscores the importance of establishing university-industry collaborations to create a trustworthy environment, as it can contribute to the creation of spin-offs.

Additionally, organizational enablers would play a significant role in governing collaborative university-industry R&D programs. Fernandes et al. [21] note that collaborations between industries and universities can take various forms, including collaborative research projects, commercialization of R&D results, entrepreneurship (such as spin-off creation), and joint curriculum design and delivery. These enablers facilitate interaction and knowledge exchange between universities and industry, fostering spin-off creation.

In summary, the influence of university-industry collaborations on spin-off creation can be explained by factors such as the number and type of relationships with other companies, the size and nature of the university's social network, trust formation between partners, and the presence of organizational enablers. These mechanisms contribute to accessing new knowledge, resources, and ideas, as well as facilitating the effective flow of knowledge, all of which are essential for spin-off creation.

The study's findings demonstrate that previous collaborations between the same University-Industry dyad or repeated collaborations have a positive effect on the creation of University Spin-Offs. This is consistent with prior research that has identified previous alliance relationships as a critical driver of collaboration success. Such relationships encourage alliance partners to share their proprietary technologies for joint invention development and increase the level of trust and reputation between them [20,68].

On the one hand, previous alliance relationships between universities and industry partners have been found to encourage the sharing of proprietary technologies for joint invention development [95]. This collaboration allows for the exchange of knowledge and resources, which can contribute to the creation of new spin-off companies that leverage the combined expertise and intellectual property of both parties involved. On the other hand, again trust plays a crucial role in the success of these collaborations by facilitating effective communication, knowledge sharing, and cooperation [96]. When trust is established between partners, it becomes easier to overcome communication barriers and work together towards shared objectives. These findings challenge the evidence that suggests communication barriers between partners are insignificant [97] and instead suggest that overcoming these barriers can facilitate future collaborations. This is in line with recent research that highlights the importance of trust and reputation in facilitating collaboration between universities and industry partners [35,98].

Taken together, these mechanisms suggest that previous collaborations between universities and industry partners have a positive impact on the creation of university spin-offs. By promoting knowledge sharing, trust building, and reputation development, these collaborations create an environment that is conducive to innovation and entrepreneurship. It is imperative for universities and industry partners to actively engage in shared governance and consider the flexibility and transparency of intellectual property (IP) policies to enhance trust and facilitate successful collaborations. The establishment of robust relationships and the cultivation of a trustworthy environment can foster a culture of collaboration, driving the creation of university spin-offs and promoting technology transfer and innovation management in the industry.

In the field of entrepreneurship this research also has some support. Networks improve the ability of entrepreneurs in the recognition of business opportunities [59,88,99]. Consequently, some studies indicate that the number of links in an entrepreneur's network is positively related to the number of business ideas and opportunities that are recognized [100]. Also, increasing the number of collaborations with different companies, will increase the network of the entrepreneurs and it would prove the necessary core base to attract the necessary capitals to stablish the SpinOff venture [101]. For example, industry collaboration during the training period of PhD students, increase the probability for them to become entrepreneurs [53]. Consequently, the more networks an academic entrepreneur has with industry, the more valuable their participation becomes for the establishment of a university Spin-Off [102].

Besides, collaborations with universities provide companies with access to a pool of talented individuals, including researchers, scientists, and students, who can contribute fresh ideas, perspectives, and skills to the innovation process [103,104]. Universities serve as hotbeds of innovation and entrepreneurship, nurturing a culture that encourages the exploration of new ideas and the creation of spin-off ventures based on cutting-edge research [17,105]. By partnering with universities, companies can tap into this entrepreneurial ecosystem and potentially benefit from the commercialization of spin-offs.

Furthermore, the presence of collaborations between universities and industry has been found to contribute to the growth of spin-offs in the post-creation stage [106]. Research indicates that academic entrepreneurs with extensive networks in industry experience faster spin-off growth. This underscores the significance of establishing and nurturing collaborations with various companies to support the progress and prosperity of spin-off ventures.

The mechanisms underlying the influence of repeated university-industry collaborations on spin-off creation can be explained through the absorptive capacity. Absorptive capacity refers to a firm's ability to identify, assimilate, and effectively utilize new external knowledge for commercial purposes [107]. It is considered a critical factor in a firm's capacity to leverage external knowledge for enhancing innovation performance. Repeated university-industry collaborations offer valuable opportunities for knowledge exchange and transfer between academia and industry, enabling academic entrepreneurs to enhance their absorptive capacity and gain access to valuable resources and expertise [108]. This enhanced absorptive capacity can positively impact firm innovation performance and contribute to the successful establishment and growth of spin-offs.

However, it is recognized that according to the results, although the effect of the number of collaborations of the university with different firms has a lower effect than prior collaborations, nonetheless having a greater number of links to the industry is considered equally important, since social, professional, and exchange relationships with other actors allow both the acquisition of important resources and the identification of opportunities in the process of creating the Spin-Off. Therefore, it is evident that universities that create a greater number of Spin-Offs are those that develop more collaborative networks, but more important is the understanding that the university would not develop prior collaborations if first do not have an exploration of potential allies from industry.

This study contributes to the ongoing debate on whether University-Industry collaborations should be promoted to enhance innovation and the creation of university Spin-Offs. The data obtained and analyzed in this study suggest that prior University-Industry collaborations would be a high valuable factor that helps to decrease the information asymmetry between the market knowledge in a university, and the research knowledge in the firm. The lack of reiterative interactions with industry makes commercialization of R&D less likely, versus contexts where efficiency is increased by developing a greater number of links and reiterative interactions with firms.

The results of this study provide evidence that university-industry collaborations play a significant role in the creation of university Spin-Offs. The findings suggest that a higher interaction of the university with different companies, and previous collaborations between the same university-company dyad increases the probability of university Spin-Off creation. These results can inform policy decisions related to technology transfer and commercialization in universities, by encouraging more collaboration between universities and industry partners. By focusing on the factors that influence the creation of university Spin-Offs, particularly in the study of University-Industry collaborations, this research could help to design a framework for policy creators in universities, firms, and agencies that promote R&D and innovation. These policies should oversee the development of effective knowledge transfer mechanisms, which facilitate and encourage the generation of the necessary skills for researchers and entrepreneurs to integrate the worlds of R&D and the market.

However, this study also has some limitations that offer opportunities for future research. For instance, future studies could focus on investigating the generalizability of these results in different countries and regions [109], as well as exploring the different types of collaborations that are necessary at different stages of the Spin-Off creation process. Furthermore, it would be valuable to investigate the role of inter-organizational networks in the creation of university Spin-Offs, considering variables such as network structure, formation, types of links, connectedness, distance, and similarity. Future research can also focus on how the strength of university-firm collaborations may depend on the number and diversity of interactions with different companies and how this contributes to the successful creation of university Spin-Offs. Also, it would be of interest to investigate the role of intermediary agents in the interface of University-Industry interactions, for example Technology Transfer Offices, or science, technology, and innovation parks. Finally, to enhance the methodological rigor of future studies, it is strongly recommended to move beyond the limitations of a cross-sectional design and adopt panel data methods. By doing so, researchers can delve deeper into the relationships between variables and conduct a more comprehensive analysis of causality. Panel data methods allow for the examination of time variations at the individual subject level, enabling a detailed assessment of the impact of university-industry collaborations on spin-off creation. Furthermore, employing these methods facilitates the exploration of how the propensity of a university to create spin-offs in the past influences the positive outcomes resulting from such collaborations. This approach will provide valuable insights into the dynamics of university-industry interactions and their effects on spin-off development.

Overall, this study provides new insight into the importance of university-industry collaborations in the creation of university Spin-Offs. The results of this study can inform policy decisions related to technology transfer and commercialization in universities, by encouraging more collaboration between universities and industry partners. Future research in this area can further improve our understanding of the key determinants of university Spin-Off creation and contribute to the development of effective strategies for commercializing university research.

## Author contribution statement

Hugo Martínez-Ardila: Conceived and designed the experiments; Analyzed and interpreted the data; Wrote the paper. Ángela Castro-Rodrigueza: Conceived and designed the experiments; Performed the experiments; Analyzed and interpreted the data; Contributed reagents, materials, analysis tools or data. Jaime Camacho-Pico: Performed the experiments; Analyzed and interpreted the data.

## Data availability statement

Data will be made available on request.

## Declaration of competing interest

The authors declare that they have no known competing financial interests or personal relationships that could have appeared to influence the work reported in this paper.
